# A Scientometric Review of Resource Recycling Industry

**DOI:** 10.3390/ijerph16234654

**Published:** 2019-11-22

**Authors:** Minxi Wang, Ping Liu, Zhaoliang Gu, Hong Cheng, Xin Li

**Affiliations:** College of Management Science, Chengdu University of Technology, Chengdu 610059, China; wangminxi@mail.cdut.edu.cn (M.W.); liupingcdu@163.com (P.L.); chenghong@cdut.cn (H.C.)

**Keywords:** waste management, resource recycling industry, green and sustainable development, circular economy, visualization analysis, CiteSpace

## Abstract

With rapid economic development and urbanization, a large number of primary resources are consumed and accumulate in society as recyclable resource, which causes great pressure on the environment. The development of the resource recycling industry (RRI) can reduce environmental impacts and achieve sustainable development and green growth. Scholars are paying more attention to the resource recycling industry (RRI), and the related literature continues to increase. There are over 7041 publications covering RRI in the Web of Science database from 1996 to 2018. This paper analyzes the time distribution characteristics of the literature and the status of the scientific research cooperation network using the visualization analysis software CiteSpace. The number of documents increased from 94 in 1996 to a peak of 963 in 2018. There is no relatively stable core author group. The number of papers published by “Chinese Acad Sci” ranks first among all research institutions. Document co-citation analysis and burst detection are adopted to assess the status and emerging trends in the RRI research domain. A publication by M.C. Monte on waste management is the most cited paper. Additionally, “green and sustainable and technology” and “science and technology—other topics” are the latest emerging subject categories in RRI research. Furthermore, “e-waste”, “reverse logistics” and “lean manufacturing” are emerging research trends for RRI, and “carbon emissions”, “policy”, “demolition waste”, “supply chain management” and “compressive strength” have become hot topics. These findings may provide inspiration for scholars to search for new research directions and ideas.

## 1. Introduction

The term “recyclable resource” in this paper is defined as “various wastes produced in the process of social production and consumption, which have lost all or part of their original use-value, and can be recycled and processed so that they can regain their use-value” [1]. Recyclable resource includes scrap metals; electronic scrap products; scrap mechanical and electrical equipment and their components; scrap paper raw materials (such as wastepaper, cotton); scrap light chemical raw materials (such as rubber, plastics, pesticide packaging, animal bone and hair) and scrap glass [1]. The resource recycling industry (RRI) refers to the enterprises engaged in recycling, processing, and utilization of recyclable resource, scientific and technological development, information services, commodity circulation of recyclable resource and other activities.

With rapid economic development and urbanization, large quantities of primary resources are consumed and accumulate in society as recycled resources, which cause great pressure on the environment. The continuous development of human society requires the acquisition of various resources from nature, and the world faces the threat of resource depletion. At the same time, the various wastes (recyclable resource) produced by humans have not been used to their maximum efficiency, which further aggravates the impact of human society on the natural environment. Vigorous development of RRI will reduce societal demand for natural resources, achieve recycling and sustainable development of resources, and thus minimize the environmental impact caused by human demands for resources from nature. Therefore, the degree of development of the RRI will be another important indicator for assessing the impact of human social development on the environment. This has also aroused widespread concern among scholars. Ongondo et al. conducted a comprehensive analysis of the management of e-waste covering many countries and regions around the world and discussed the future direction of e-waste [2]. Xu et al. reviewed the global status of waste solar panel recycling [3]. Jefferson Hopewell et al. reviewed plastic recycling and presented challenges and opportunities for plastic recycling [4]. Chen et al. reviewed the development status of the inhibition of the anaerobic digestion process [5]. Additionally, Yao and Zhang [6] systematically sorted out and analyzed the policies of China’s RRI to study the impact of policies on the industry. However, these studies are conducted from the single perspective of the RRI, such as waste recycling, recycling technologies, environmental impacts and industrial policies.

**Figure 1:**
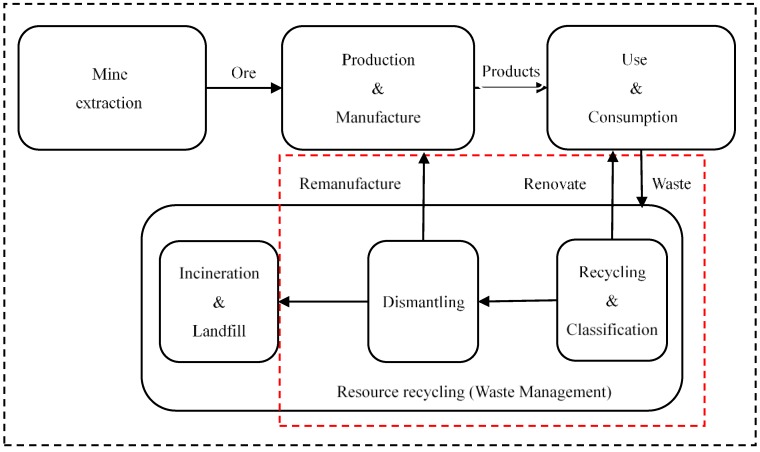
System boundaries of the resource recycling industry visualization analysis.

According to the theories of material flow analysis and life cycle analysis, we can divide the flow of resources into four stages, as shown in Figure 1. The four phases are mine extraction; production and manufacture; use and consumption and resource recycling (waste management). In this paper, we focus on the fourth stage, and the system boundary of the study is indicated by the red dotted line in Figure 1. Based on Figure 1 and the theory of material flow analysis, the final hosts of waste should be two. The first host is to return to society after remanufacturing or renovation, and the second host is to incinerate or place in landfills, thereby returning to the natural world. However, incineration and landfills cause serious environmental pollution. For the sake of minimizing the environmental impact of waste generated by society, we must promote the development of RRI to improve the resource recycling rate. There are still many problems in the development of RRI. Exploring the status quo and development trends of RRI research, and promoting the deepening of related research will contribute to the sustainable and healthy development of the RRI. This paper is based on 7041 articles in the core collection database of Web of Science (WOS) from 1996 to 2018. We used the literature measurement visualization software, CiteSpace, combined with social network analysis, co-citation analysis, emergent analysis, information science and bibliometrics, and the research results of the RRI are systematically sorted out. Therefore, the research path and knowledge clustering of the RRI are identified, and the research hotspots and evolutionary contexts are explored. The results obtained from this study can help new researchers to quickly understand the research status of the RRI field so that they can devote themselves to research within a short period of time. These findings may provide inspiration for scholars to search for new research directions and ideas. In the end, it will promote the deepening development of related research on RRI and promote the ecological development of RRI.

## 2. Methodology and Data Sources

At present, the most common and authoritative scientific databases in the world are the Web of Science (WOS), Scopus and Google Scholar. Many scholars have also conducted a detailed comparative analysis of the data coverage of WOS, Scopus and Google Scholar, and of the quality of journals and the advantages and disadvantages of these databases [7,8,9].

Wang and Waltman [10] conducted a comparative analysis of Scopus and Web of Science in the article “Large-scale analysis of the accuracy of the journal classification systems of Web of Science and Scopus”. It turns out that according to their citation-based criteria Web of Science performs significantly better than Scopus in terms of the accuracy of its journal classification system. What is more, the WOS database provides more consistent and standardized documentation of the literature in comparison to other databases, including the title of the paper, abstracts, keywords, article types, journals, year of publication, volume number, page number and references [11,12]. These records are necessary for visualization and bibliometric analysis. Moreover, Falagas et al. found that the literature data obtained from the WOS database was able to produce better visualizations [13]. In the summary analysis of previous scientific measurement articles, we also learned that most scholars tend to use the WOS database when performing bibliometric and visualization analysis [14,15,16,17,18,19]. Additionally, when using CiteSpace for visualization analysis, the literature data obtained from WOS can use all of its analysis functions, and the data obtained from the Scopus database cannot use all of these functions, such as domain co-occurrence analysis [20]. For the above reasons, this paper chose to use the WOS database to search the literature data. To ensure the quality of the literature data, we chose WOS’s core collection database, including SCI-Expanded, SSCI, CCR-Expanded and IC. The data in this article comes from the Web of Science database, so the statistical methods for articles published by authors, institutions and countries are the same.

**Figure 2:**
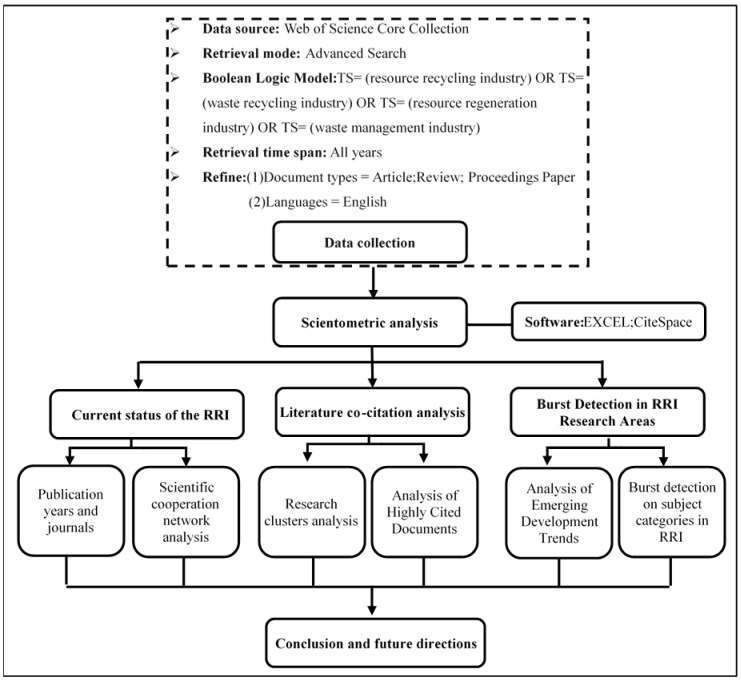
Research design.

According to the system boundary of the research content of this paper, as shown in Figure 1, we first chose the resource recycling industry as the search keyword. Considering that different countries or different scholars use different terms to represent the resource recycling industry, we tried different keywords to search the literature during the search process. We then compared the correlation between the literature searched with different keywords and the research topic of this paper and consulted experts in related fields. The data acquisition method and the scientometric analysis structure of this paper is shown in Figure 2. We determined the following search strategy: TS = (resource recycling industry) OR TS = (waste recycling industry) OR TS = (resource regeneration industry) OR TS = (waste management industry), Timespan = “All years”; articles and reviews are selected as literature types. Among them, TS = Topic, which is a search expression used for advanced retrieval in the Web of Science database. For example, TS = (resource recycling industry), which means find records of literature containing the terms resource recycling industry in the Topic field. Then, a manual screening step was performed to remove the articles that did not match the theme, and 7041 articles were finally obtained. The literature search and download date was December 28 2018. It should be noted that the literature data analyzed in this paper did not include “grey literature” such as research reports. The main reason is that the “grey literature” does not have the standard recording format required for visualization analysis. This is also a common problem faced by scholars when using CiteSpace for visualization analysis. However, previous research papers indicate that this does not have much impact on the results of the visualization analysis [21].

The documentation visualization analysis tool is CiteSpace software, and the software version is CiteSpace 5.3. R4. The software used to draw the histogram of the number of documents is EXCEL. CiteSpace is translated as “citation space”. It is multi-dimensional, time-sharing and dynamic visualization analysis software developed by Chaomei Chen, Professor of Computer and Information Science at Drexel University in the United States and is based on the JAVA language. Burst detection can be used to detect abrupt changes in nodes, including author, country, keywords and more [22]. Betweenness centrality in CiteSpace is also a measure of the importance of nodes in the network. In CiteSpace’s visualization map, key nodes that link different domains usually have high betweenness centrality [23]. CiteSpace software can be used to understand the structure, regularity and distribution of a certain knowledge domain, it can conduct collaborative analysis by authors, institutions and countries to explore the changing laws of a scientific field. It is also possible to find the knowledge base, research trends, research hotspots and frontiers in a scientific field. In recent years, CiteSpace has received extensive attention from scholars as efficient and powerful scientometric software [24]. Due to the many risks related to information security, Li and Li [25] analyzed the knowledge structure and the future direction of information security by using CiteSpace. Using CiteSpace, Xiao et al. explored the development status of organic photovoltaic technology and the trends of future research [26]. Wei et al. studied the geographic information systems knowledge domain and current research hotspots [27]. Yu and Chao [28] conducted a co-citation analysis of related research on carbon emissions trading and explored its subject categories, main research fields and new emerging trends. It should be noted that some of the visualization analysis techniques and bibliometric analysis methods used in this article are the same as were used in the above articles. It also shows that the analysis methods used in this paper are practiced by scholars and are considered to be scientific.

## 3. Results and Discussion

### 3.1. Current Status of the Resource Recycling Industry (RRI)

#### 3.1.1. Publication Years and Journals

**Figure 3:**
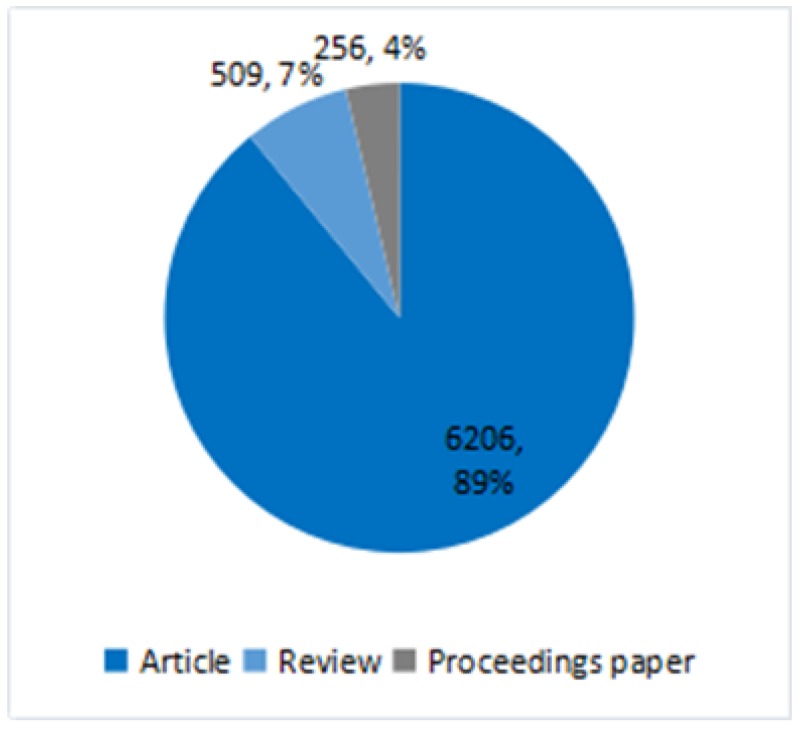
The distribution of document types.

**Figure 4:**
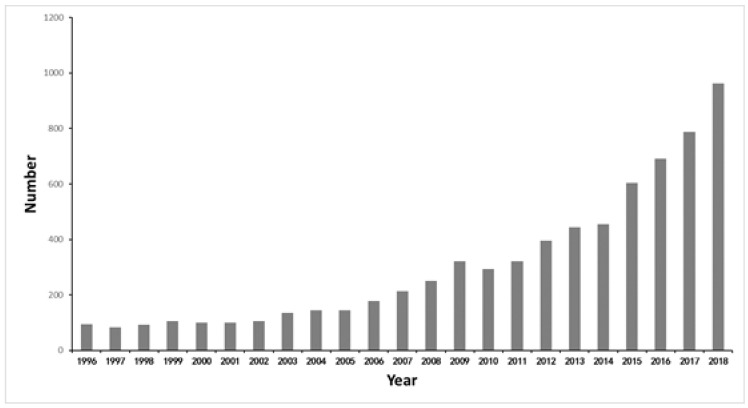
The number of publications in the resource recycling industry (RRI) area from 1996 to 2018.

The publication of academic papers is generally considered to be an indicator for measuring the level of development of a discipline. The change in the number of documents can directly reflect changes in the amount of scientific knowledge [29]. According to Figure 3, the document types mainly concentrate on three categories: article (6206), review (509) and proceedings papers (256). This paper counted the number of documents retrieved using Excel software and created a histogram of the document distribution, as shown in Figure 4. During the past decades, the number of documents regarding RRI had increased steadily from 94 in 1996 to a peak of 963 in 2018. From another perspective, related research in the resource recycling industry has attracted more attention from society and from scholars. Additionally, according to the growth rate of the number of documents, we could divide the literature quantity levels into two stages. The first stage is from 1996 to 2009, where the growth in the number of documents was relatively slow. At this stage, scholars gradually began to pay attention to the field of RRI. The second stage is from 2009 to 2018, and the growth rate of literature quantity has increased. Although the Copenhagen World Climate Conference, held in 2009, failed to produce a legally binding agreement, it aroused wide concern and discussion of global climate change and environmental issues in the international community. This may be one of the most important factors in 2009 as the turning point of literature quantity change.

From the search results, we could see that more than one hundred journals published research papers related to the resource recycling industry. We selected the top ten journals with the largest number of published articles and created Table 1. As shown in Table 1, the Journal of Cleaner Production is the journal with the most published papers relevant to this study. A total of 614 papers were published in the Journal of Cleaner Production, accounting for 8.72% of the total published papers. The impact factor of the Journal of Cleaner Production is 5.651, and the subject categories “Engineering, Environmental” and “Renewable and Sustainable Energy Reviews” have the highest impact factor among the 10 journals studied, with an impact factor of 9.184, and the subject category is “Green and Sustainable Science and Technology”. The subject categories of these journals are mainly about environmental science, engineering technology, biotechnology, microbiology, architecture and sustainable science. This shows that research papers in the field of RRI involve multidisciplinary fields.

#### 3.1.2. Scientific Cooperation Network Analysis

CiteSpace mainly provides three levels of scientific cooperation network analysis, namely, the micro-author cooperation network (co-Author), the meso-institutional cooperation network (co-institution) and macro-national cooperation (co-country/territory). Katz and Martin define scientific cooperation as the research of scholars who work together to create new scientific knowledge [30]. In actual scientific research, scientific cooperation manifests itself in a variety of forms. The scientific cooperation referred to in this paper was that there were different authors, institutions or countries for the same article. We could believe that there was a cooperative relationship between these authors, institutions and countries.

##### Co-Author Analysis

A core research scholar in a field of research can be found through statistical analysis of authors. Table 2 lists the top 20 authors who published the greatest number of articles. From Table 2, we found that LI JH was the author with the most published articles with a total of 26. Price’s law is one of the laws of scientometrics, which comes from Derek J. de sola price’s “small science, big science” (Davis, 1985; Price, 1963) [31]. Price’s law is generally used to describe the quantitative relationship between the number of scientists and the number of scientific literature, as well as between scientists of different ability levels. According to Price’s law, the core author’s certification formula is M ≈ 0.749√Nmax. In the formula, Nmax is the author with the largest number of posts; M is the minimum number of posts by the core author. In this paper, M ≈ 0.749 × 5.099 = 3.81. Therefore, authors who have published a large number of four articles can be identified as core authors. According to the statistical results, a total of 132 scholars published more than four papers, and the 132 core authors published a total of 854 papers. Since the number of articles published by core authors is less than 50% of the total, it can be considered that there is no relatively stable core author group in the RRI field.

**Figure 5:**
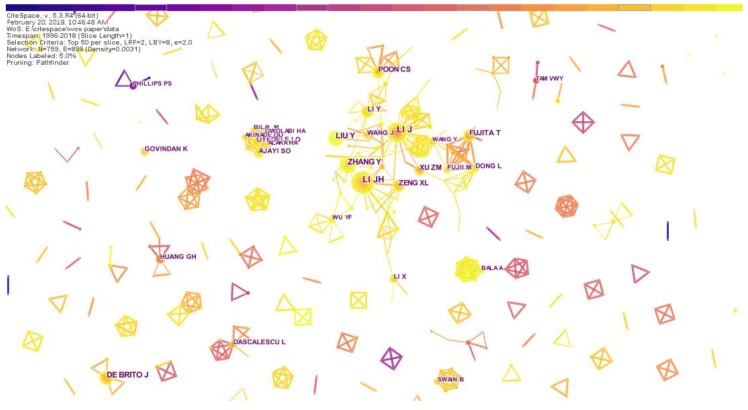
Network map showing author cooperation.

We then imported 7041 documents retrieved from the Web of Science into CiteSpace. In the author’s collaborative network analysis, the timespan selected from 1996 to 2018, the time slice selected was 1 year, and the selection criteria were the top 50%. Figure 5 shows an author’s cooperative network map of research in the field of resource recycling industry. In Figure 5, the size of a node represents the number of papers published by the author. The map shows the time distance in cool and warm tones, from cool to warm, indicating time from far and near. As seen from the parameters in the upper left corner of the spectrum, the density of the network was 0.0031. In general, the nodes were relatively scattered and there were fewer connections between nodes. That shows that, although RRI researchers have a certain scale, they are scattered, and there are many relatively isolated authors, only some of the more closely related research teams. Among them, the cooperative group centered on authors such as LI JH, ZHANG Y, LI J and LIU Y, and this group was the largest and most closely connected.

##### Co-Institution Analysis

According to the statistical results, a total of 454 institutions around the world have published research papers related to the resource recycling industry. We selected the top ten institutions (by number of papers published) and plotted the results in Table 3.

**Figure 6:**
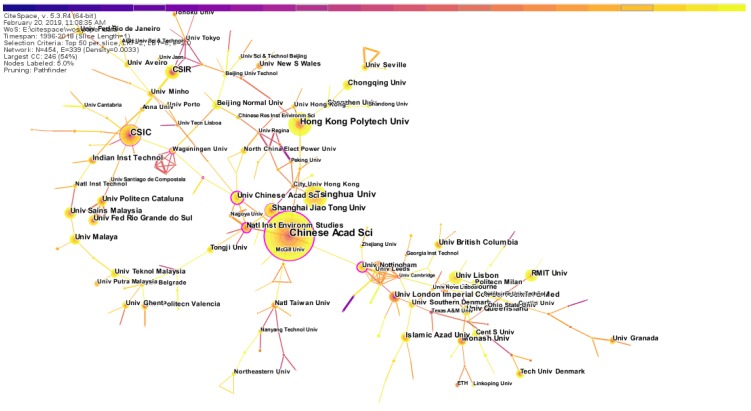
Network map of institutional cooperation.

From Table 3, the number of RRI-related research papers published by the Chinese Academy of Sciences was the greatest, with a total of 112 papers published. Among the top ten institutions by the number of papers published, in second and third place were Hong Kong Polytechnic University and Tsinghua University. Overall, among the top ten institutions (ranked by the number of published papers), four were from China. England, Spain, Australia, Portugal, India and Malaysia each have one institution in the top ten. USA, Germany, Italy, Canada and Brazil have no research institutions in the top ten, although the number of papers published in these countries was relatively large. Figure 6 shows the institution’s cooperative network map of research on the field of RRI. The overall tone of the institutional cooperation network map was warm, and the connecting lines were also mostly yellow. This shows that exchanges and cooperation between institutions have recently been closed. There may be two main reasons: first, the rapid development of computer and information technology, international exchange and cooperation are more convenient; second, the in-depth development of renewable resources industry research, scholars from different disciplines and different fields are cooperating. In the institutional cooperation network map, the “Chinese Acad Sci” has a purple outer ring outside the node, indicating that it has high betweenness centrality. Therefore, it can be considered that “Chinese Acad Sci” plays an important role in research cooperation between institutions.

##### Co-Country/Territory Analysis

Different countries have paid different amounts of attention to RRI research. We extracted the top ten countries by the number of published papers. From Table 4, we see that China was the country that had published the most research papers in RRI related fields. China was followed by the USA, England, Spain, India, Australia, Brazil, Germany, Italy and Canada. Among the top ten, three were developing countries, namely, China, India and Brazil. This shows that although developing countries had a certain sharing role in RRI research, the dominant countries were still the developed countries.

**Figure 7:**
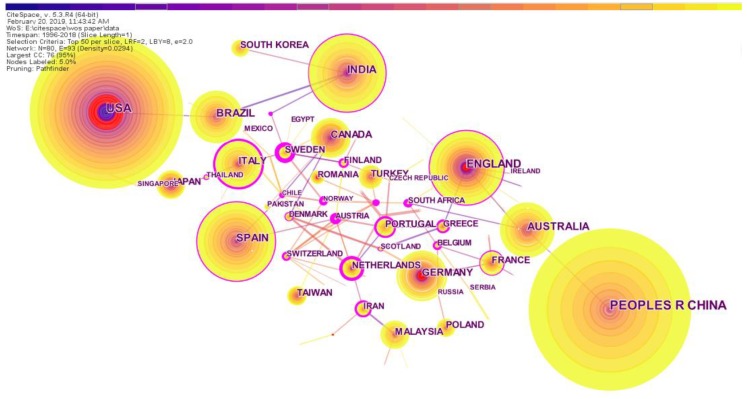
Network map of country cooperation.

Figure 7 shows the national cooperation network, and there were a total of 80 nodes and 93 lines, which means 80 countries were involved in cooperation. In Figure 7, “PEOPLES R CHINA” is a shorthand for the “People’s Republic of China” in the Web of Science database. In the text of this paper, we still used the more commonly used word “China” for the sake of brevity. Among them, there were more lines between the developed countries of Europe, indicating that their research cooperation and exchanges were more closely related. From the centrality, Italy had the highest central value, with a central value of 0.23. This can be illustrated by the fact that the Italy node had a purple outer ring. England ranked second with a central value of 0.19. In this case, developed countries such as Italy and England can greatly influence research trends in this area. The map shows the time distance in cool and warm tones, from cool to warm, indicating time from far and near. Similarly, the different colors in the nodes indicate the far and near time of the country’s published literature. There were more cool colors in the USA nodes than in China, which reflects that the USA research in the RRI field started earlier than China. Besides, there was a red circle in each of the nodes of the USA, England and Germany, which was the result of the detection of the burst value. A country had a burst value, indicating that the country’s number of publications in the RRI field had increased rapidly during a certain period of time. Although China’s tree rings were slightly larger than those for the United States, China’s ring layers were fewer than for the United States. This means that the number of publications in the People’s Republic of China had increased dramatically in recent years, but that early research in the United States had a solid foundation. In a way, we could view China as a powerful force in RRI research and that the United States had always been the leading force. Overall, the development of the RRI field was inseparable from the strong cooperation of various countries in the world.

### 3.2. Literature Co-Citation Analysis

Mutual citation in the scientific literature indicates that scientific literature is not isolated but is a system of mutual connections and continuous extension. The mutual references between scientific literature reflect the accumulation, continuity, and inheritance of scientific knowledge. Co-citation analysis means that two documents appear together in the bibliography of a third citing document so that the two papers can be considered to form a co-citation relationship [32]. It is generally believed that highly-cited literature constitutes a source of the knowledge base in a subject area, and highly cited authors are also considered to have greater influence in the field of their research. Therefore, using the CiteSpace’s function of co-citation analysis, we could discover the knowledge base, key literature and main research areas for resource recycling industry research.

#### 3.2.1. Research Clusters Analysis

**Figure 8:**
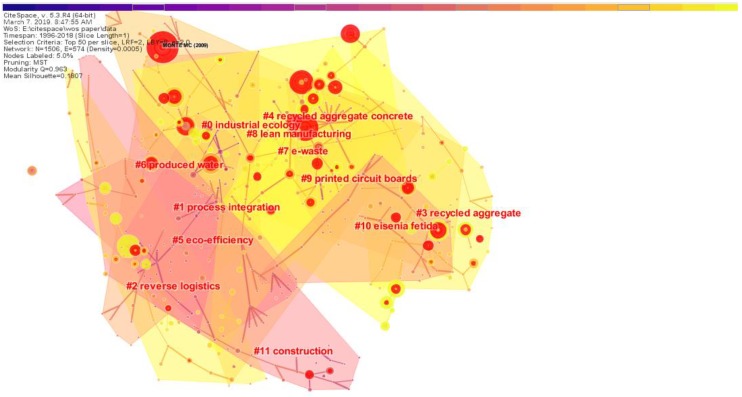
Cluster network for the RRI research area.

In the literature co-citation analysis, the relevant parameters of the CiteSpace software were set as follows: the time width used was from 1996 to 2018, the time slice selection was 1, the screening standard was top50, and the network cutting mode selected MST. The minimum spanning tree (MST) is a network clipping algorithm that improves the readability of the network by preserving important connections in the network. The idea of the MST algorithm is to construct a spanning tree containing the smallest sum of all vertices and weights based on the original graph G (Chen, 2006) [23]. As shown in Figure 8, 11 major clusters formed after running the software. In addition, the red nodes in the figure were documents with high bursts. After data analysis, we generated Table 1, showing the top ten largest research clusters. In Table 1, size represents the number of members included in each cluster. Silhouette is an indicator to evaluate the clusters. Specifically, the clustering was evaluated by measuring the indicators of network homogeneity. The closer the value of the silhouette is to 1, the higher the homogeneity of the network. A Silhouette value >0.5 means that the clustering result is rational. In this section, the log-likelihood ratio algorithm (LLR) was used to label the clusters. The log-likelihood ratio algorithm (LLR) is a method used by Professor Dunning to extract similar terms in text and calculate the similarity rate to name the cluster (Dunning, 1993) [33]. In Table 1, the last column of mean represents the average of the reference years. This represents the average year in which the literature was published in the same cluster. It can be used to judge the old and new work in a document cluster and is very useful for researchers.

From Table 5, cluster #5 “eco-efficiency” is the earliest clustering in the RRI field. This shows that the reason scholars first studied RRI is because of the excellent ecological benefits and positive effect on environmental protection and resource conservation. In addition, we know that Cluster 7, Cluster 2 and Cluster 8 are newly formed Clusters, which means that “e-waste”, “reverse logistics” and “lean manufacturing” are the hot spots in recent RRI research. With the development of society, e-waste poses one of the world’s greatest pollution problems [34]. Therefore, scientific research on the recycling of electronic waste is particularly important. Reverse logistics is defined as “The process of planning, implementing, and controlling the efficient, cost-effective flow of raw materials, in-process inventory, finished goods and related information from the point of consumption to the point of origin for the purpose of recapturing value or proper disposal” [35]. Reverse logistics plays a vital role in the construction of resource recycling network systems. After the collection of scrapped products from customers, repair, dismantling, remanufacturing, recycling and other methods are adopted [36]. It should be noted that lean manufacturing here means that the product should follow the principle of reduction when it is produced. This means using fewer raw materials and energy inputs to achieve the intended production or consumption purposes then save resources and reduce pollution from the sources of economic activities. At the same time, in production, the principle of reduction often appears to require a lean product.

#### 3.2.2. Analysis of Highly Cited Documents

We sorted out the basic information for the papers that were the top ten most cited, as shown in Table 6. It must be noted that the number of citations referred to in this article did not refer to the number of citations from the WOS but the number of citations among the 7041 documents retrieved for this article. This was derived from CiteSpace’s literature co-citation analysis function. It indicates that “Waste management from pulp and paper production in the European Union” published by M.C. Monte on Waste Management is the most cited paper among the 7041 documents. Brett H. Robinson analyzed and predicted the current and future global production of electrical waste. He also analyzed potential environmental contaminants associated with e-waste and studied environmental pollution caused by e-waste during recycling and disposal. He believes that e-waste in today’s society has become ubiquitous and that pollution caused by e-waste has caused considerable pollution to the environments of developing countries and has already threatened human health [37]. F.O. Ongondo conducted in-depth research and analysis on e-waste management practices and made some critical comments. In addition, he also made some suggestions and prospects for the generation, governance and supervision of e-waste [2]. Additionally, Cui and Zhang [38] published a review of the recovery of metals from electronic waste. He presented the initial research on the topic and discussed the mechanisms and models of biosorption of precious metal ions from solutions. S.M. Al-Salem retrospectively analyzed the recycling and recycling routes of plastic solid waste [39]. Patrizia Ghisellini provided a review of the literature of the last two decades and analyzed the main circular economy features and perspectives: origins, basic principles, advantages and disadvantages at different levels [40]. From the above, among the ten highly cited papers, three concern e-waste and three concern techniques and methods for resource recovery and disposal. It can be seen that e-waste and resource recycling are important research contents in the RRI field.

### 3.3. Burst Detection in RRI Research Areas

In CiteSpace, the algorithm proposed by Kleinberg, J. in 2002 was used for burst detection [46]. According to the choice of burst nodes, it can be divided into burst topics, documents, authors, journals and fields. In CiteSpace, the more burst nodes a cluster contains, the more active the field is or the more active are the emerging trends of research [47].

#### 3.3.1. Analysis of Emerging Development Trends

When we performed burst detection, if the type of a node in the visual map was an article, we usually considered that these articles with citation bursts had received special attention from the academic community in a past period. In addition, if a research cluster contains many articles with bursts, then we could consider this research cluster to be an emerging trend [48]. After we analyzed the data with CiteSpace, we found that there were many documents with citation bursts. We listed the top 10 articles with citation bursts, as shown in Table 7.

It should be explained that the entire line in the last column of Table 7, Table 8 and Table 9 represents the period of the study (1996–2018), and the red part represents the period of a citation burst. Among them, one of the dotted lines (-) represents one year. According to the ranking burst, the first was MONTE MC [41], with a burst value of 12.868 and a cluster number of #0. The second was ROBINSON BH [37] with a burst value of 10.271 and a cluster number of #7. The third was AL-SALEM SM [39] with a burst value of 10.062 and a cluster number of #12. The 4th was BINNEMANS K [45] with a burst value of 9.259 and a cluster number of #7. The 5th was PARFITT J [49] with a burst value of 9.125 and a cluster number of #8. The 6th was FINNVEDEN G [42] with a burst value of 9.080 and a cluster number of #6. The 7th was Narayanan and ONGONDO FO [2] with a burst value of 8.498 and a cluster number of #9. The 8th was HOPEWELL J [4] with a burst value of 8.113 and a cluster number of #12. The 9th was RECK BK [50] with a burst value of 8.093 and a cluster number of #7. The 10th was GRAEDEL TE [43] with a burst value of 7.095 and a cluster number of #7. After the above analysis, we clearly knew that there were four papers in the top ten high citation bursts references that belonged to cluster #7. This also reflects, from another perspective, that “e-waste” is an emerging research trend in the field of RRI research. Among the top 10 references, the top-ranked item by bursts was MONTE MC in Cluster #0, which means that “industrial ecology” is an important research area.

Additionally, with a keyword burst detection analysis of CiteSpace software, we could explore the rapidly growing topics in this field [48]. Through detailed analysis using CiteSpace, we found a number of keywords with bursts and selected the top 20 keywords for these bursts; see Table 8. The changes in the burst keywords in the list can be roughly divided into three phases according to time. The first phase was 1996–1999, the second phase was 2000–2009 and the third phase was 2010–2018. In the first phase, the main purpose of RRI development was to achieve “pollution prevention”, “waste minimization” and “sustainable development”. The methods of waste treatment at that time mainly included “incineration”, “landfill”, “recycle” and “reuse”. The most prominent concern at the time was “hazardous waste”. In the second phase, the relatively prominent goal of RRI research was to achieve “industrial ecology”. The main methods adopted were “cleaner production” and “solid waste management”. This stage was focused on “industrial waste”. In the third phase, with the deepening of scientific research, research content had become extensive and detailed. At this stage, the research by international scholars focused on “carbon emissions”, “reverse logistics”, “policy”, “e-waste”, “demolition waste”, “supply chain management” and “compressive strength”. At the same time, the research topics at this stage were also emerging research trends in the RRI field. The above research shows that the research theme of the resource recycling industry was constantly changing over time.

#### 3.3.2. Burst Detection on Subject Categories in RRI Research Area

**Figure 9:**
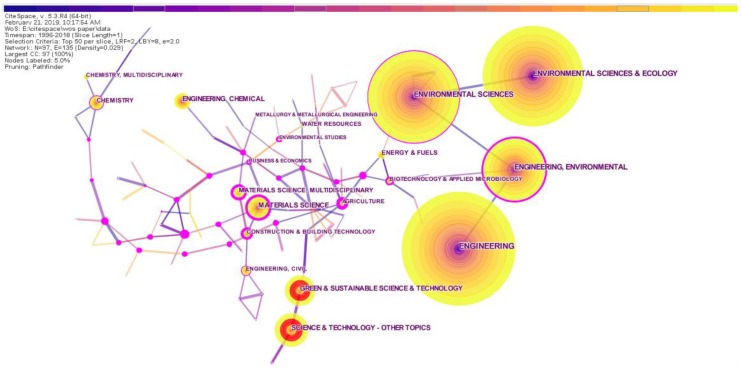
Co-occurrence network of subject categories.

When using CiteSpace for data analysis, the node type selects the category, and after running the software, the co-occurrence network of the subject categories in the RRI field could be obtained, as shown in Figure 9. As seen from this figure, RRI research involves engineering, environmental science and ecology, materials science, chemistry, energy science, architecture, applied microbiology, polymer science, agricultural science, biotechnology, metallurgical technology and sustainable science. Among these, engineering, environmental science and ecology and environmental science have the highest frequencies. In CiteSpace, a node with a red inner ring represents a burst node. Figure 9 shows only the two nodes with the highest bursts, namely, “GREEN and SUSTAINABLE and TECHNOLOGY” and “SCIENCE and TECHNOLOGY—OTHER TOPICS”. Subsequently, we conducted burst detection of subject categories and listed the top ten subject categories, as shown in Table 9. These results show that “GREEN and SUSTAINABLE and TECHNOLOGY” and “SCIENCE and TECHNOLOGY—OTHER TOPICS” were also the latest emerging subject categories of RRI research.

## 4. Conclusions

In this paper, 7041 papers retrieved from the Web of Science database were used as the data foundation, and research on the resource recycling industry was visualized and analyzed. Some analysis results were as follows.

First, during the past decades, the number of documents covering RRI increased steadily from 94 in 1996 to a peak of 963 in 2018, which fully explained that the research in the resource recycling industry had attracted more attention from society and from scholars. The journal “Journal of Cleaner Production” was the journal with the most publications. Among the top 10 most productive journals, the journal “Renewable and Sustainable Energy Reviews” had the highest impact factor.

Second, it could be considered that there was no relatively stable core author group in the RRI field. The number of RRI-related research papers published by the Chinese Academy of Sciences was the greatest. The number of publications in the People’s Republic of China had increased dramatically in recent years, but early research in the United States had provided a solid foundation. Additionally, international exchanges and cooperation in RRI field research mainly involve developed countries.

Third, “Waste management from pulp and paper production in the European Union” published by M.C. Monte on waste management was the most cited paper among the 7041 documents. “GREEN and SUSTAINABLE and TECHNOLOGY” and “SCIENCE and TECHNOLOGY—OTHER TOPICS” were the latest emerging subject categories of RRI research. Furthermore, “e-waste”, “reverse logistics” and “lean manufacturing” were RRI emerging research trends, and “carbon emissions”, “policy”, “demolition waste”, “supply chain management” and “compressive strength” had become hot topics.

In general, research in the field of resource recycling industry mainly focused on specific waste recycling (such as “electronic waste”), environmental impact (“carbon emissions”), policy and technical aspects. In the future, research around specific recyclable resources will remain a more active area for many years to come. In addition, researching the resource recycling industry from the perspective of industrial ecosystem coordination may be the direction that scholars should pay attention to in future research.

## Figures and Tables

**Table 1:** The top 10 journals in terms of publications.

Journal	Number of Publications	Percentage of Total Publications	Impact Factor	Categories
Journal of Cleaner Production	614	8.72	5.651	Engineering, Environmental
Resources Conservation and Recycling	301	4.27	5.120	Engineering, Environmental
Waste Management	227	3.22	4.723	Engineering, Environmental
Construction and Building Materials	159	2.25	3.485	Construction and Building Technology
Journal of Environment Management	103	1.46	4.449	Environmental Science
Journal of Chemical Technology and Biotechnology	102	1.44	2.587	Biotechnology and Applied Microbiology
Waste Management and Research	100	1.42	1.955	Engineering, Environmental
Sustainability	90	1.28	2.075	Green and Sustainable Science and Technology
Journal of Hazardous Materials	88	1.24	6.434	Engineering, Environmental
Renewable and Sustainable Energy Reviews	73	1.03	9.184	Green and Sustainable Science and Technology

**Table 2:** The top 20 authors in the RRI research area.

Code	Author	Quantity	Centrality	Code	Author	Quantity	Centrality
1	LI JH	26	0.01	11	XU ZM	13	0.00
2	LI J	24	0.02	12	OYEDELE LO	12	0.00
3	ZHANG Y	19	0.01	13	AJAYI SO	12	0.00
4	LIU Y	17	0.00	14	DASCALESCU L	11	0.00
5	DE BRITO J	17	0.00	15	LI X	11	0.00
6	POON CS	16	0.01	16	HUANG GH	11	0.00
7	FUJITA T	15	0.00	17	GOVINDAN K	10	0.00
8	LI Y	15	0.01	18	FUJII M	10	0.00
9	ZENG XL	14	0.00	19	WANG J	10	0.00
10	FRIAS M	13	0.00	20	DONG L	10	0.00

**Table 3:** The top 10 institutions in terms of publications.

Code	Institutions	Number of Publications	Percentage of Total Publications	Centrality
1	Chinese Academy of Science (China)	112	1.59	0.27
2	Hong Kong Polytechnic University (China)	55	0.78	0.08
3	Tsinghua University (China)	55	0.78	0.00
4	Superior Council of Scientific Investigations (Spain)	50	0.71	0.16
5	Council of Scientific industrial research (India)	37	0.52	0.08
6	Shanghai Jiao Tong University (China)	36	0.51	0.15
7	University of Lisbon (Portugal)	32	0.45	0.01
8	University of Science (Malaysia)	31	0.44	0.01
9	Imperial College of Science, Technology, and Medicine (England)	31	0.44	0.05
10	RMIT University (Australia)	30	0.42	0.01

**Table 4:** The top 10 institutions in terms of publications.

Code	Country	Frequency	Percent/%	Burst	Centrality
1	PEOPLES R CHINA	945	13.4	-	0.00
2	USA	899	12.8	30.72	0.00
3	ENGLAND	480	6.8	22.05	0.19
4	SPAIN	470	6.7	-	0.13
5	INDIA	468	6.6	-	0.09
6	AUSTRALIA	374	5.3	-	0.10
7	BRAZIL	194	4.9	-	0.10
8	GERMANY	335	4.7	22.42	0.09
9	ITALY	316	4.5	-	0.23
10	CANADA	296	4.2	-	0.08

**Table 5:** The largest 10 clusters in the RRI research area.

Cluster ID	Size	Silhouette	Label (LLR)	Mean (Year)
0	43	0.666	industrial ecology	2006
1	40	0.875	process integration	2005
2	38	0.816	reverse logistics	2010
3	38	0.737	recycled aggregate	2009
4	37	0.79	recycled aggregate concrete	2007
5	37	0.837	eco-efficiency	2002
6	36	0.628	produced water	2006
7	34	0.634	e-waste	2012
8	33	0.667	lean manufacturing	2010
9	33	0.697	printed circuit boards	2008

**Table 6:** The top 10 cited documents.

Frequency	Centrality	Author	Year	Journal	Literature
40	0.01	M.C. Monte	2009	Waste Management	[41]
32	0.03	Brett H. Robinson	2009	Science of The Total Environment	[37]
30	0.00	S.M. Al-Salem	2009	Waste Management	[39]
28	0.00	Patrizia Ghisellini	2016	Industrial Ecology	[40]
27	0.00	F.O. Ongondo	2011	Waste Management	[2]
26	0.02	Goran Finnveden	2009	Journal of Environmental Management	[42]
22	0.01	T. E. Graedel	2011	Journal of Industrial Ecology	[43]
21	0.01	S. Marinkovic	2010	Waste Management	[44]
21	0.10	Jirang Cui	2008	Journal of Hazardous Materials	[38]
19	0.00	Koen Binnemans	2013	Journal of Cleaner Production	[45]

**Table 7:** The top 10 references with the strongest citation bursts.

References (DOI)	Cluster ID	Year	Strength	Begin	End	1996–2018
MONTE MC, 2009, WASTE MANAGE, V29, P293	0	2009	12.868	2012	2018	**-----------------------**
ROBINSON BH, 2009, SCI TOTAL ENVIRON, V408, P183	7	2009	10.271	2012	2018	**-----------------------**
AL-SALEM SM, 2009, WASTE MANAGE, V29, P2625	12	2009	10.062	2015	2018	**-----------------------**
BINNEMANS K, 2013, J CLEAN PROD, V51, P1	7	2013	9.259	2016	2018	**-----------------------**
PARFITT J, 2010, PHILOS T R SOC B, V365, P3065	8	2010	9.125	2016	2018	**------------------------**
FINNVEDEN G, 2009, J ENVIRON MANAGE, V91, P1	6	2009	9.080	2013	2016	**-----------------------**
ONGONDO FO, 2011, WASTE MANAGE, V31, P714	9	2011	8.498	2015	2018	**-----------------------**
HOPEWELL J, 2009, PHILOS T R SOC B, V364, P2115	12	2009	8.113	2016	2018	**-----------------------**
RECK BK, 2012, SCIENCE, V337, P690	7	2012	8.093	2014	2018	**-----------------------**
GRAEDEL TE, 2011, J IND ECOL, V15, P355	7	2011	7.095	2015	2018	**-----------------------**

**Table 8:** The top 10 references with the strongest citation bursts.

Keywords	Strength	Begin	End	1996–2018
Waste minimization	7.219	1996	2006	**-----------------------**
Incineration	5.246	1996	2008	**-----------------------**
Pollution prevention	8.420	1996	2008	**-----------------------**
Hazardous waste	6.734	1997	2011	**-----------------------**
Landfill	4.901	1997	2004	**-----------------------**
Sustainable development	3.522	1998	2002	**----------------------**
Waste management	5.457	1998	2002	**----------------------**
Reuse	6.676	1999	2009	**-----------------------**
Recycle	8.698	1999	2008	**-----------------------**
Industrial waste	6.976	2000	2010	**----------------------**
Industrial ecology	13.333	2005	2013	**-----------------------**
Solid waste management	8.123	2006	2011	**-----------------------**
Cleaner production	8.282	2007	2013	**-----------------------**
Carbon emission	4.182	2010	2015	**-----------------------**
Reverse logistics	8.152	2013	2015	**-----------------------**
Policy	5.329	2013	2015	**-----------------------**
Electronic waste	13.212	2013	2018	**-----------------------**
Demolition waste	16.6.7	2014	2016	**-----------------------**
Supply chain management	8.607	2015	2018	**-----------------------**
Compressive strength	13.129	2016	2018	**-----------------------**

**Table 9:** The top 10 subject categories with bursts.

Subject categories	Strength	Begin	End	1996–2018
MATERIALS SCIENCE, TEXTILES	10.921	1996	2005	**-----------------------**
METALLURGY & METALLURGICAL ENGINEERING	17.456	1996	2005	**-----------------------**
WATER RESOURCES	15.937	1996	2007	**-----------------------**
MATERIALS SCIENCE, PAPER& WOOD	14.701	1996	2001	**-----------------------**
POLYMER SCIENCE	14.525	1996	2003	**-----------------------**
MATERIALS SCIENCE	13.939	1996	2000	**-----------------------**
NUCLEAR SCIENCE & TECHNOLOGY	26.776	1996	2006	**-----------------------**
FISHERIES	10.940	2001	2010	**-----------------------**
SCIENCE & TECHNOLOGY-OTHER TOPICS	32.275	2016	2018	**-----------------------**
GREEN & SUSTAINABLE SCIENCE &TECHNOLOGY	44.858	2016	2018	**-----------------------**
